# Time-lagged and acute impact of heat stress on production and fertility traits in the local dual-purpose cattle breed “Rotes Höhenvieh” under pasture-based conditions

**DOI:** 10.1093/tas/txaa148

**Published:** 2020-08-05

**Authors:** Kathrin Halli, Kerstin Brügemann, Mehdi Bohlouli, Sven König

**Affiliations:** Institute of Animal Breeding and Genetics, Group Animal Breeding, Justus-Liebig-University, Giessen, Germany

**Keywords:** birth weight, calving interval, dual-purpose cattle, heat stress, stillbirth, weight development

## Abstract

Climate change causes rising temperatures and extreme weather events worldwide, with possible detrimental time-lagged and acute impact on production and functional traits of cattle kept in outdoor production systems. The aim of the present study was to infer the influence of mean daily temperature humidity index (mTHI) and number of heat stress days (nHS) from different recording periods on birth weight (BWT), 200 d- and 365 d-weight gain (200 dg, 365 dg) of calves, and on the probability of stillbirth (SB), and calving interval (CINT) of their dams. Data recording included 4,362 observations for BWT, 3,136 observations for 200 dg, 2,502 observations for 365 dg, 9,293 observations for the birth status, and 2,811 observations for CINT of the local dual-purpose cattle breed “Rotes Höhenvieh” (RHV). Trait responses on mTHI and nHS were studied via generalized linear mixed model applications with identity link functions for Gaussian traits (BWT, 200 dg, 365 dg, CINT) and logit link functions for binary SB. High mTHI and high nHS before autumn births had strongest detrimental impact on BWT across all antepartum- (a.p.) periods (34.4 ± 0.79 kg maximum). Prolonged CINT was observed when cows suffered heat stress (HS) before or after spring calvings, with maximum length of 391.6 ± 3.82 d (56 d a.p.-period). High mTHI and high nHS during the 42 d- and 56 d a.p.-period implied increased probabilities for SB. We found a significant (*P* < 0.05) seasonal effect on SB in model 3 across all a.p.-periods, with the highest probability in autumn (maximum of 5.4 ± 0.82% in the 7 d a.p.-period). Weight gains of calves (200 dg and 365 dg) showed strongest HS response for mTHI and nHS measurements from the long-term postnatal periods (42 d- and 56 d-periods), with minimum 200 dg of 194.2 ± 4.15 kg (nHS of 31 to 42 d in the 42 d-period) or minimum 365 dg of 323.8 ± 3.82 kg (mTHI ≥ 60 in the 42 d-period). Calves born in summer, combined with high mTHI or high nHS pre- or postnatal, had lower weight gains, compared with calves born in other calving seasons or under cooler conditions. Highest BWT, weight gains, and shortest CINT mostly were detected under cool to moderate climate conditions for mTHI, and small to moderate nHS. Results indicate acute and time-lagged HS effects and address possible HS-induced epigenetic modifications of the bovine genome across generations and limited acclimatization processes to heat, especially when heat occurs during the cooler spring and autumn months.

## INTRODUCTION

In the future, Germany will more frequently be affected by extreme weather events including anomalies in daily maximum temperature (Deutschlaender and Delalane, 2012), with direct impact on primary and functional cattle traits. In recent years, heat stress (HS) studies mainly focused on performance, health, and fertility traits of high-yielding dairy cows from modern large-scale herds. An arising field of scientific interest addresses the effect of time-lagged HS in dairy cows.

Especially the last 2 mo of gestation are important for bovine fetus growth, as 60% of weight gain is attributed to this period (Baumann and Currie, 1980). Against this background, HS during late gestation in dams (described in the literature as “maternal HS” [e.g., Monteiro et al., 2016b; Tao et al., 2012]) negatively influenced performance traits of offspring during the first year of aging (Monteiro et al., 2016a). Direct or acute HS (i.e., HS with immediate impact on animal traits) caused reduced daily weight gains in growing crossbred calves (Habeeb et al., 2011) and feedlot heifers (Mitlöhner et al., 2001). Regarding reproduction traits, HS during insemination contributed to significantly greater stillbirth rates and longer calving intervals in Holstein cows (El-Tarabany and El-Tarabany, 2015).

So far, limited research from the perspective of acute and time-lagged HS influence addressed local beef or dual-purpose cattle kept in pasture-based production systems. Adaptation mechanisms from local cattle breeds might differ from trait responses to environmental challenges as identified in high-yielding Holstein Friesian cows. In Germany, the “Rotes Höhenvieh” cattle breed (RHV) is of increasing importance, because RHV cattle are used for quality beef production in low input grazing systems. The overall breeding goal for RHV only comprises low heritability fertility traits, which are usually sensitive to environmental changes (Dash et al., 2016). However, due to specific RHV breed characteristics, HS response might differ as known from other beef or dairy breeds.

In consequence, the present study aimed on inferring time-lagged and direct effects of HS during several recording periods around parturition on production and female fertility traits in the local endangered dual-purpose breed RHV, considering an across-generation approach.

## MATERIALS AND METHODS

The research did not involve any direct physical contact to the animals, and no experimental studies were conducted for this project. Therefore, no additional statement of institutional animal care and use committee is required.

### Animal Traits

Rotes Höhenvieh is a medium-sized and single-colored red-brown cattle breed with horns and hard dark claws. The RHV cattle are modest and robust, and in consequence, RHV is a breed suggested for grazing systems, even in challenging habitats with low fodder energy, etc. Rotes Höhenvieh originally was developed for three purposes: milk production, meat production, and for field work. However, in the past decades, due to the focus on indoor cattle production systems and consideration of specialized breeds for either milk or meat production, the RHV population gradually declined. All data were provided by “Vereinigte Informationssysteme Tierhaltung w. V.” (VIT) (Verden, Germany), with permission of all RHV breeder societies involved. The RHV data set included the performance traits birth weight (BWT) (from 162 herds), 200 d-weight gain (200dg) (from 130 herds), 365 d-weight gain (365dg) (from 137 herds), the female fertility trait calving interval (CINT) (from 132 herds), and birth status reports (living or stillborn calf) (from 124 herds). Stillbirth (SB) was defined as calves born dead or calves that died within the first 48 h after birth. The RHV herds are spread over Germany and represent pasture-based production systems. The grazing season is from the end of April until the beginning of November. In the remaining winter months, cattle are kept indoors. On pasture, apart from the natural provision of shade (trees, bushes, etc.), no specific HS management is applied.

All traits were corrected for outliers. Accordingly, calves with a 200dg < 70 kg, calves with a 365dg < 140 kg or > 475 kg or cows with a CINT < 297 d or > 512 d were excluded from the ongoing analyses. Herds representing only one birth status record, or herds that had announced only stillborn or only live born calves, were excluded. Two hundred d-weight gain and 365dg only were considered from animals with both trait measurements from the same herd. Data recording spanned a period of 20 years (2000 to 2019) and included 4,362 observations for BWT, 3,136 observations for 200dg, 2,502 observations for 365dg, 9,293 observations for the birth status (SB or living calf), and 2,811 observations for CINT. Lactation number of cows was grouped into 8 parity groups, where all cows with lactation numbers > 8 were included in parity group “8.” Calving seasons were defined as “winter” (December, January, February), “spring” (March, April, May), “summer” (June, July, August), and “autumn” (September, October, November).

### Meteorological Data

According to the postal codes, herds were assigned to geographic regions and merged with the nearest official weather station. In this regard, longitude and latitude information was used for the identification of the nearest weather station, using the Geosphere package version 1.5-10 in *R* (Hijmans et al., 2019). For the analyses, 45 different weather stations were allocated to the farms. The mean distance between a farm and a weather station was 22.7 km (minimal distance: 523.4 m; maximal distance: 54.5 km).

The temperature humidity index (THI) was calculated using the following equation:

$$\begin{array}{ll} THI= & \left[\left(1.8\times T\;{}^{\circ }C\right)+32\right]-\left[0.55\left(0.0055\times RH\%\right)\right]\\ & \times \left[\left(1.8\times T\;{}^{\circ }C\right)-26\right], \end{array}$$

where T is the dry bulb temperature and RH is the relative humidity (NRC,1971).

A mean daily THI (mTHI) was calculated for different recording periods before and after birth of the calf for the calf production traits (BWT, 200dg, 365dg), or before and after calving (a.p., p.p.) for female fertility traits (SB, CINT). In this regard, we considered three recording periods: 7, 42, and 56 d before and after the trait recording date (according to Muller et al., 1975; Baumann and Currie, 1980; Monteiro et al., 2016a). Four mTHI-classes were defined as follows: class 1: mTHI ≤ 39, class 2: mTHI 40 to 49, class 3: mTHI 50 to 59, and class 4: mTHI ≥ 60. Furthermore, we counted and classified the numbers of days representing HS (nHS), i.e., the number of days where mTHI was ≥ 60. Classes of nHS within the different recording periods were defined as follows:

$$\begin{array}{l} \begin{array}{ll} & 7\;d-period:\;class\;1:\;0\;\;to\;\;2\;d;\;class\;2:\;3\;\;to\;\;5\;d;\\ & \;class\;3:\;6\;to\;7\;d\\ & 42\;d-period:\;class\;1:\;0\;to\;10\;d;\;class\;2:\;11\;to\;20\;d;\\ & \;class\;3:\;21\;\;to\;\;30\;\;d;\;class\;4:\;31\;to\;42\;d\\ & 56\;d-period:\;class\;1:\;0\;to\;10\;d;\;class\;2:\;11\;to\;20\;\;d;\\ & \;class\;3:\;21\;to\;30\;d;\;class\;4:\;31\;\;to\;\;40\;d;\\ & \;class\;5:\;41\;\;to\;\;56\;d. \end{array} \end{array}$$

**Fig. 1.**
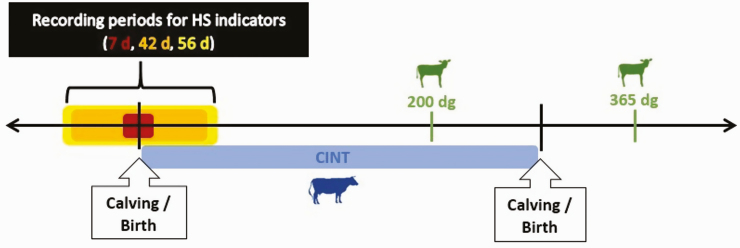
Illustration of recording periods for the HS indicators mean daily temperature humidity index (mTHI) and number of heat stress days (nHS), considered for 200 d-weight gain (200dg), 365 d-weight gain (365dg), and for calving interval (CINT).

**Fig. 2.**
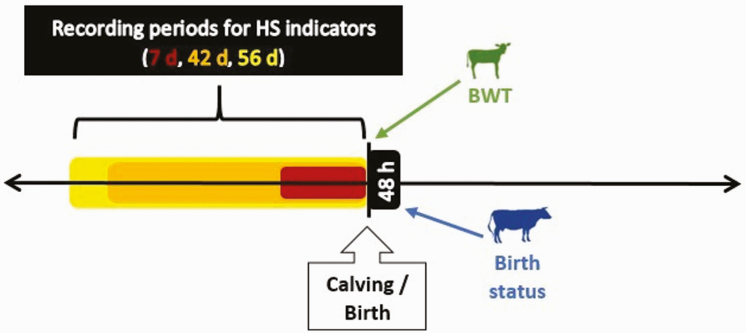
Illustration of recording periods for the HS indicators mean daily temperature humidity index (mTHI) and number of heat stress days (nHS), considered for birth weight (BWT) and for birth status.

The traits 200 d-weight gain, 365dg, and CINT were analyzed for effects of mTHI and nHS, calculated for all pre- and postnatal/a.p.- and p.p.-periods (Fig. 1). Birth weight and birth status were analyzed for effects of mTHI and nHS, calculated for all prenatal or a.p.-periods, only (Fig. 2).

### Statistical Models

For statistical analysis, linear mixed models and generalized linear mixed models as implemented in the SAS University Edition (SAS Institute, Cary, NC) were applied to infer associations between climatic effects (mTHI-class or nHS-class) with calf production traits and female fertility traits. In this regard, we focused on two modeling approaches. Models 1 and 2 considered the HS indicators nested within seasonal effects, whereas models 3 and 4 considered HS indicators and season as separate fixed effects. The first model was defined to analyze the impact of mTHI from different recording periods on BWT, 200dg, and on 365dg. The respective model 1 was as follows:

$$\begin{array}{l} y_{ijklmn}=\;\mu +\;c_{i}+\;p_{j}+\;f_{k}+\;g_{m}+\;s{\left(c\right)}_{li}+\;e_{ijklmn}, \end{array}$$ [1]

where *y* are the observations for BWT, 200dg, or 365dg; *µ* is the overall mean effect; *c*_*i*_ is the fixed effect of mTHI-class (1, 2, 3, and 4) at the 7 d-, 42 d-, or 56 d-period; *p*_*j*_ is the fixed effect of parity group (1, 2, 3, 4, 5, 6, 7, and ≥ 8); *f*_*k*_ is the fixed effect of herd (1 to 162); *g*_*m*_ is the fixed effect of sex (male or female); *s*(*c*)_*li*_ is the fixed effect of mTHI during the time periods nested within calving season (winter, spring, summer, autumn); and *e*_*ijklmn*_ is the random residual effect.

To analyze the impact of nHS on BWT, 200dg, and 365dg, model 2 was defined as follows:

$$\begin{array}{l} y_{ijklmn}=\;\mu +\;c_{i}+\;p_{j}+\;f_{k}+\;g_{m}+\;s{\left(c\right)}_{li}+\;e_{ijklmn}, \end{array}$$ [2]

where *y* are the observations for BWT, 200dg, or 365dg; *µ* is the overall mean effect; *c*_*i*_ is the fixed effect of nHS-class (1, 2, 3, 4, and 5) at the 7 d-, 42 d-, or 56 d-period; *p*_*j*_ is the fixed effect of parity group (1, 2, 3, 4, 5, 6, 7, and ≥ 8); *f*_*k*_ is the fixed effect of herd (1 to 162); *g*_*m*_ is the fixed effect of sex (male or female); *s(c)*_*li*_ is the fixed effect of nHS-class during the time periods nested within calving season (winter, spring, summer, and autumn); and *e*_*ijklmn*_ is the random residual effect.

To analyze the impact of mTHI or nHS on CINT, the applied models were similar to models 1 and 2; however, the fixed effect of sex was not included.

Generalized linear mixed models with a logit-link function were applied to study the influence of climatic effects mTHI (model 3) and nHS (model 4) on binary SB. The respective models were as follows:

$$\begin{array}{l} Logit\left(p_{ijklm}\right)\;=\;\mu \;+\;c_{i}+\;p_{j}+\;s_{k}+\;f_{l}+\;a_{m}, \end{array}$$ [3]

where *p*_*ijklm*_ is the probability of a stillbirth occurence; *µ* is the overall mean effect; *c*_*i*_ is the fixed effect of mTHI-class (1, 2, 3, and 4) at the 7 d-, 42 d-, or 56 d a.p.-period; *p*_*j*_ is the fixed effect of parity group (1, 2, 3, 4, 5, 6, 7, and ≥ 8); *s*_*k*_ is the fixed effect of calving season (winter, spring, summer, and autumn); *f*_*l*_ is the random effect of herd (1 to 124); and *a*_*m*_ is the random effect of the cow,

and

$$\begin{array}{l} Logit\left(p_{ijklm}\right)\;=\;\mu \;+\;c_{i}+\;p_{j}+\;s_{k}+\;f_{l}+\;a_{m}, \end{array}$$ [4]

where *p*_*ijklm*_ is the probability of a stillbirth occurence; *µ* is the overall mean effect; *c*_*i*_ is the fixed effect of nHS-class (1, 2, 3, 4, and 5) at the 7 d-, 42 d-, or 56 d a.p.-period; *p*_*j*_ is the fixed effect of parity group (1, 2, 3, 4, 5, 6, 7, and ≥ 8); *s*_*k*_ is the fixed effect of calving season (winter, spring, summer, and autumn); *f*_*l*_ is the random effect of herd (1 to 124); and *a*_*m*_ is the random effect of the cow.

For all models, the threshold for testing the significance of fixed effects was *P* < 0.05.

## RESULTS AND DISCUSSION

In the following subchapters, we focused on trait responses, which were significantly associated with the HS indicators mTHI and nHS. The THI is the most common index to estimate the level of thermal stress (Dash et al., 2016) and was applied in humans as well as in animals. Quite constant THI above the recovery threshold that lasts for several days was found to be a reinforcing factor of heat, as recovery phases are important elements of coping with heat loads (Hahn, 1999). Hence, both, mTHI and nHS are important indicators for HS, as confirmed in the present study. In this regard, a comprehensive set of results for the HS influence from all applied models considering mTHI and nHS from the different recording periods on primary and functional traits can be found in the supplemental material.

### Influence of Climatic Effects on Calf Production Traits

#### Birth weight.

The influence of the mTHI-class and the nHS-class on BWT was not significant across all prenatal recording periods (results from models 1 and 2, respectively), except for the impact of the nHS-class from the 7-d prenatal period (*P* < 0.05) (Table 1). In the 7-d prenatal period, BWT was lowest when nHS comprised the maximal number of 6 to 7 d (35.8 ± 0.61 kg) (Supplementary Table S1). Accordingly, mTHI ≥ 60 for the last 6 and 8 wk of pregnancy were associated with lowered BWT (42 d-period: 36.2 ± 0.26 kg; 56 d-period: 36.3 ± 0.25 kg). Furthermore, least-squares means (LSMeans) for BWT decreased when nHS exceeded 30 d in the 56 d-period before birth. In Holstein dairy cattle, seasonal effects on BWT were identified, i.e., higher average weights for calves born during cooler months of the year (Linden et al., 2009). Effects were partly time-lagged, indicating possible in utero HS on birth weights of dairy calves. Confirming this, Monteiro et al. (2016a) identified reduced BWT and lower weights up to 1 yr of age in calves from dams that suffered HS during the last 6 wk of gestation. Accordingly, our results support the time-lagged effects of in utero HS on BWT in the RHV dual-purpose breed, especially during the critical time of fetus development. Explanations for time-lagged HS-effects are as follows: 1) a HS-induced reduction in gestation length by 4 d (Tao et al., 2012), 2) a HS-induced placental insufficiency with intrauterine growth retardation (Yates et al., 2011), and 3) fetal hyperthermia, caused by the increasing body temperature of the dam (Faurie et al., 2001). Some physiological traits of the dam are known to be affected by HS and are presumably directly linked to birth weight of calves. In dairy cows, HS reduced feed intake (Ominski et al., 2002), as well as feeding and rumination times (Halli, 2018). In primiparous cows, a low level of nutrition during the last third of gestation affected BWT of offspring (Drennan, 1979). Consequently, suboptimal maternal body condition due to a HS-induced reduction of feed intake might contribute to lower BWT in offspring.

**Table 1.** Table of significances: Effects of mTHI-class or nHS-class and effects of nested mTHI-class or nested nHS-class within calving season during different recording periods on production and fertility traits

	Recording period^*a*^					
	7 d		42 d		56 d	
	a.p.^*b*^	p.p.^*c*^	a.p.	p.p.	a.p.	p.p.
Trait^*d*^	mTHI-class^*e*^					
BWT, kg	Ns^*f*^		ns		ns	
200dg, kg	*	ns	ns	*	ns	***
365dg, kg	ns	*	*	*	ns	**
CINT, d	ns	ns	ns	ns	ns	ns
SB, %	ns		ns		ns	
	nHS-class^*g*^					
BWT, kg	*		ns		ns	
200dg, kg	**	ns	*	***	ns	***
365dg, kg	**	**	***	***	**	***
CINT, d	ns	ns	**	ns	***	ns
SB, %	ns		ns		ns	
	nested mTHI-class within calving season					
BWT, kg	**		***		***	
200dg, kg	***	***	***	***	***	***
365dg, kg	***	***	***	***	***	***
CINT, d	***	***	***	***	***	***
	nested nHS-class within calving season					
BWT, kg	**		***		***	
200dg, kg	***	***	***	***	***	***
365dg, kg	***	***	***	***	***	***
CINT, d	***	***	*	***	*	***

^*a*^Recording periods: 7 d = 7 day period; 42 d = 42 day period; 56 d = 56 day period

^*b*^a.p. = ante partum / prenatal.

^*c*^p.p. = post partum / postnatal.

^*d*^Traits: BWT = birth weight; 200dg = 200 d-weight gain; 365dg = 365 d-weight gain; CINT = calving interval; SB = probability for stillbirth.

^*e*^mTHI = mean daily temperature humidity index.

^*f*^ns = not significant.

^*g*^nHS = number of heat stress days.

*P < 0.05; **P < 0.01; ***P < 0.001.

In the current study, also a significant impact on calf BWT was found for the fixed effect of the nested mTHI-class (model 1) and the nested nHS-class (model 2) within calving season for all prenatal periods (*P* < 0.01). Birth weight was lowest when mTHI was ≥ 60 before autumn-births in all recording periods. Also in autumn, high nHS caused lowest BWT in calves (e.g., 34.4 ± 0.79 kg (6 to 7 heat stress [HS] days during the 7 d-period)) (Supplementary Table S2). Impact of the fixed effect of the nested mTHI-class and the nested nHS-class within calving season on BWT signalizes a greater sensitivity for heat and for frequently appearing HS-conditions during the generally cooler autumn months. A lower pasture quality and feed availability at the end of summer also contributes to lower BWT.

#### 200 d-weight gain.

A significant influence of mTHI before birth on 200dg (model 1) was only observed for the 7 d-period (*P* < 0.05), where highest mTHI (≥ 60) was associated with lowest 200dg (202.4 ± 3.76 kg) (Supplementary Table S1). Dairy cows, who suffered HS during the dry period, responded with a milk yield decline up to 30 wk of lactation, compared with cows, which were cooled for the entire dry period (Fabris et al., 2019). Especially in suckler cows, a HS-induced reduction in milk yield might contribute to lower weight gains of their calves. Monteiro et al. (2016b) further hypothesized that maternal HS during late pregnancy epigenetically modifies the fetal genome, resulting in metabolically and immunologically inefficiency in offspring. Direct impact of HS on DNA methylation patterns was proven in pigs and guinea pigs (Hao et al., 2016; Weyrich et al., 2016). In cattle embryos, a possible effect of HS on gene expression was confirmed during the pre-implantation stage (Sakatani, 2017). With regard to the postnatal periods for meteorological data and 200dg, mTHI ≥ 60 from the 7 d-period (203.9 ± 4.41 kg) and in the 56 d-period (201.6 ± 3.19 kg) also caused lowest weight gains. The impact was highly significant for the 56 d-period (*P* < 0.001) (Supplementary Table S1). Stress effects of direct heat exposure on weight gains were proven, with significantly reduced daily weight gains, in crossbred calves (Habeeb et al., 2011) and in feedlot heifers (Mitlöhner et al., 2001). In the present study, 200dg was considerably lower for calves suffering HS in the 56 d-period after birth, possibly due to a HS-induced decline in feed intake (Ominski et al., 2002).

The production trait 200dg of calves was significantly influenced by nHS in the 7 d-period (*P* < 0.01) and in the 42 d-period before birth (*P* < 0.05) (model 2). A large number of heat days (6 to 7 heat days in the 7 d-period and 21 to 30 heat days in the 42 d-period) caused lowest 200dg (179.1 ± 10.52 kg and 185.3 ± 17.14 kg, respectively) (Supplementary Table S1). In addition, the number of heat days from the 42 d- and 56 d-period after birth had a significant effect on 200dg of calves (*P* < 0.001). When nHS was highest, 200dg of calves was lowest. This was the case for all postnatal periods (e.g., 194.2 ± 4.15 kg [42 d-period]).

**Fig. 3.**
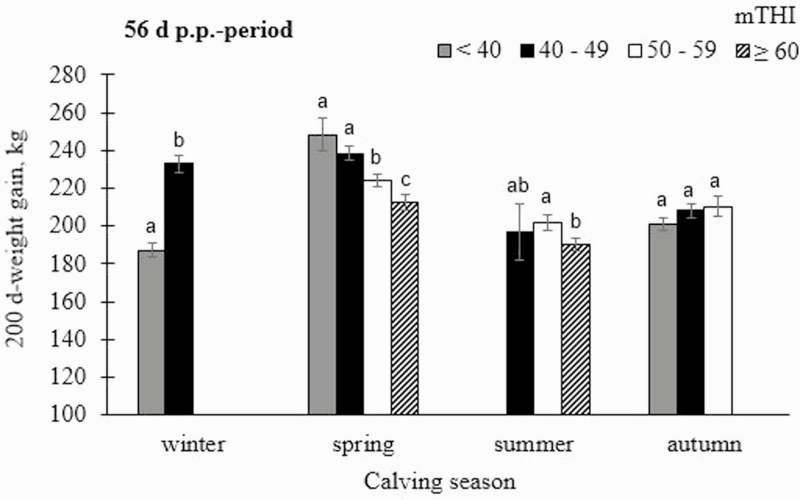
Least-squares means (LSMeans) with corresponding SE for 200 d-weight gain (200dg) in dependency of mean daily temperature humidity index (mTHI), nested within calving season from the postnatal 56 d-period. Significances of pairwise comparisons are indicated with different letters (a–c) (*P* < 0.05).

The fixed effect of nested mTHI-class within calving season was significant across all pre- and postnatal periods (*P* < 0.001) (model 1). In this regard, 200dg was lowest when mTHI was ≥ 60 in the 7 d- and the 56 d-prenatal period before summer births (192.4 ± 3.38 and 184.6 ± 3.85 kg, respectively) (Supplementary Table S2). Time-lagged HS-effects might be due to influences in the embryonic stage, but also due to “metabolic programming,” e.g., the impact of HS on adaptive processes in response to nutritional challenges (Monteiro et al., 2016b). With regard to the postnatal periods, extreme climatic conditions, e.g., heat after summer births, had strongest detrimental impact on 200dg of calves (Fig. 3). In this regard, mTHI ≥ 60 in the 7 d-period after summer births was associated with a 200dg in calves of 189.0 ± 3.34 kg only, whereas 200dg was 206.3 ± 3.97 kg when mTHI was between 50 and 59 in the same period.

**Fig. 4.**
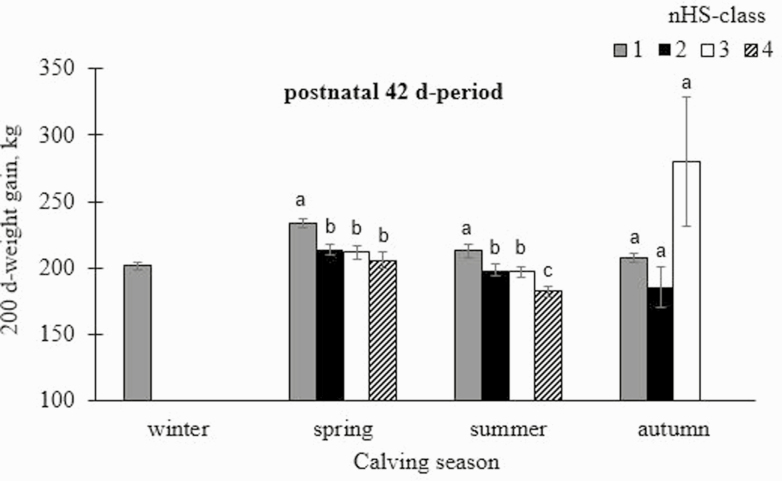
Least-squares means (LSMeans) with corresponding SE for 200 d-weight gain (200dg) in dependency of class of number of heat stress days (nHS-class) (class 1: 0 to 10 d; class 2: 11 to 20 d; class 3: 21 to 30 d; class 4: 31 to 42 d), nested within calving season from the postnatal 42 d-period. Significances of pairwise comparisons are indicated with different letters (a–c) (*P* < 0.05).

There was a significant impact of the fixed effect of nested nHS-class within calving season across all pre- and postnatal periods (*P* < 0.001) on 200dg (model 2). We also found a strong detrimental impact of the highest nHS-class before and after summer births (Supplementary Table S2). The strong detrimental effect of the combined influence of “high mTHI” or “high nHS” with the season “summer” might be due to the poorer food quality in grazing systems during the hot summer months. RHV cattle are used for low input meat production and for landscape conservation, and they are not supported with feed supplements (e.g., concentrates) during the summer months. In summer, low to moderate nHS postnatal were associated with highest 200dg. For example, a maximal nHS of 2 d in the 7 d-period caused a 200dg of 206.7 ± 4.03 kg in calves. Furthermore, when nHS ranged from 0 to 10 in the 56 d-period, calves reached a 200dg of 217.5 ± 6.10 kg (Fig. 4). Explanations address the capacity of adaptation to cope with hot environmental conditions by gradual acclimatization (Prosser, 2013), in order to minimize adverse effects on performance traits. In such context, night cooling in outdoor systems contributed to HS-compensation during a moderate number of heat days (Scott et al., 1983), indicating a short-term HS tolerance. Berman (1968) identified cows keeping their production level quite constant while their metabolic rate was 20% lower in summer compared to winter. According to Kadzere et al. (2002), sudden and prolonged heat causes a reduced capacity of acclimatization in cows. Our results suggest that physiological adaptation mechanisms are also limited in local RHV cattle, suggesting herd management improvements under pasture-based conditions.

#### 365 d-weight gain.

The impact of mTHI-class on 365dg of calves was significant for the 42 d-period before birth, only (*P* < 0.05) (model 1). For mTHI between 50 and 59 during this period, 365dg was highest (349.8 ± 9.49 kg) (Supplementary Table S1). However, for severe HS with mTHI ≥ 60, 365dg decreased (338.3 ± 4.57 kg), indicating lime-lagged HS influence only for very high temperature x humidity combinations. Across all postnatal periods, there was a significant impact of the mTHI-class on 365dg (7 d-period: *P* < 0.05; 42 d-period: *P* < 0.05; 56 d-period: *P* < 0.01) (model 1). In this regard, 365dg was lowest for mTHI ≥ 60 during the 42 d- and 56 d-period. Results indicate a stronger compromising effect of direct exposure to heat after birth on calf weight gain, compared with in utero HS before birth. Heat stress during the 42 d- and 56 d-period after birth affected 365dg similarly as it affected 200dg. Hence, calves suffering HS during the first 6 or 8 wk of life show an impaired growth potential at later ages. Accordingly, Monteiro et al. (2016a) identified reduced weights up to 1 yr of age in calves from dams that suffered HS during the last 6 wk of gestation. Significant impact of the nHS-class on 365dg of calves was found for HS influence from all prenatal periods (7 d-period: *P* < 0.01; 42 d-period: *P* < 0.001; 56 d-period: *P* < 0.01), and from all postnatal periods (7 d-period: *P* < 0.01; 42 d-period: *P* < 0.001; 56 d-period: *P* < 0.001) (Supplementary Table S1).

The fixed effect of nested mTHI-class within calving season was significant for all pre- and postnatal periods (*P* < 0.001) (model 2). With regard to heat influence from all prenatal periods, the lowest 365dg was found when mTHI was 50 to 59 before summer births (e.g., 42 d-period: 312.7 ± 4.11 kg) (Supplementary Table S2). Similarly, lowest 365dg in calves was found for high postnatal mTHI (e.g., mTHI 50 to 59 in the 7 d-period: 310.3 ± 4.84 kg) after summer births. The fixed effect of the nested nHS-class within calving season was significant for all pre- and postnatal periods (*P* < 0.001). In contrast to the obvious mTHI influence, there was no pronounced detrimental effect of high nHS-classes proven for the prenatal periods. However, with regard to the 42 d- and 56 d-period before birth, 365dg tended to be generally lower when the calf was born in summer, compared with births during the other seasons (Supplementary Table S2). In the postnatal periods, the negative effect of high nHS-classes on calf performances was very obvious. In the 42 d-period, lowest 365dg was associated with 21 to 30 heat days (315.0 ± 4.62 kg) and with 31 to 42 heat days (317.5 ± 4.78 kg) after summer births. Similarly, high nHS in the 56 d-period after summer births caused lowest 365dg (31 to 40 heat days: 310.7 ± 4.74 kg; 41 to 56 heat days: 318.1 ± 4.96 kg). Hence, results support the hypothesis that direct exposure to heat after birth affects weight development in calves stronger, compared with time-lagged in utero HS.

### Influence of Climatic Effects on Female Fertility Traits

#### Probability of stillbirth.

Across all a.p.-periods, there was no significant effect (*P* > 0.05) of the mTHI- or nHS-class on the probability of SB (models 3 and 4, respectively). Applying model 3, the probability of SB ranged from 3.5 ± 0.5% (mTHI 40 to 49 in the 7 d-period) to 5.4 ± 1.0% (mTHI ≥ 60 in the 56 d-period) (Supplementary Table S3). Applying model 4, the probability of SB ranged from 3.1 ± 0.7% (nHS 11 to 20 d in the 42 d-period) to 6.1 ± 1.3% (nHS 41 to 56 d in the 56 d-period). Stillbirth rates reflect incidences for other cattle breeds kept in outdoor production systems, such as German Angus (2.6 to 5.3%; Müllenhoff, 2008; Hohnholz Bassum, 2019) or German Fleckvieh (0.9 to 5.4%; Müllenhoff, 2008).

**Fig. 5.**
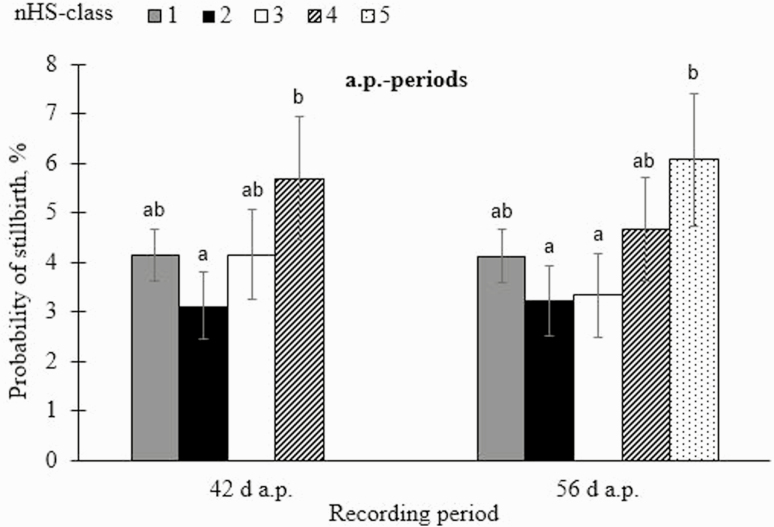
Probability of stillbirth (SB) with corresponding SE in dependency of number of heat stress days (nHS-class) from the 42 d- and 56 d-period ante partum (a.p.) (42 d-period: class 1: 0 to 10 d; class 2: 11 to 20 d; class 3: 21 to 30 d; class 4: 31 to 42 d; 56 d-period: class 1: 0 to 10 d; class 2: 11 to 20 d; class 3: 21 to 30 d; class 4: 31 to 40 d; class 5: 41 to 56 d). Significances of pairwise comparisons are indicated with different letters (a and b) (*P* < 0.05).

With regard to the 42 d- and 56 d a.p.-period, mTHI ≥ 60 caused highest probabilities of SB (5.31 ± 1.01 and 5.37 ± 1.01%, respectively), but differences among mTHI levels (results from model 3) were not significant. Similarly, stillbirth probabilities were largest (5.69 ± 1.25 and 6.07 ± 1.33%) when nHS comprised 31 to 42 days in the 42 d-period, or 41 to 56 d in the 56 d-period, respectively (results from model 4). However, applying model 4, significant (*P* < 0.05) differences were found between nHS-classes (Fig. 5). For example, a lower nHS comprising 11 to 20 d in the 42 d-period caused a probability of 3.12 ± 0.68%, only. An increase of stillbirth rates with increasing mTHI- and nHS might be due to environmentally induced stress. Accordingly, Hovingh (2009) identified an increase of abortions during a sudden increase of environmental temperatures. Avendaño-Reyes et al. (2010) identified higher rates for calving difficulties in very hot months, compared with cooler seasons. Furthermore, Monteiro et al. (2016a) reported a stillbirth rate of 4.1% in calves that suffered maternal HS during the last 6 to 7 wk of gestation, but stillbirth rates were zero for the cow group under cooling conditions during the dry period.

#### Calving interval.

Calving interval of dams was not significantly affected by the mTHI in any of the recording periods (*P* > 0.05) (model 1). However, CINT was longest when mTHI was ≥ 60 in the 7 d a.p.-period (375.4 ± 3.74 d), or for mTHI between 50 and 59 in the 42 d a.p.-period (394.9 ± 11.06 d) and in the 56 d a.p.-period (384.3 ± 8.02 d) (Supplementary Table S1). Mean THI ≥ 60 during the 56 d p.p.-period were also associated with longest CINT. The fixed effect of nHS on CINT was significant for the 42 d and 56 d a.p.-period (*P* < 0.01; *P* < 0.001) (model 2), but long nHS did not necessarily imply long CINT. In contrast to the a.p.-periods, there was no significant impact of the nHS-class on CINT across all p.p.-periods, but high numbers of heat days contributed to longer CINT (e.g. 31 to 42 d [42 d-period]: 398.0 ± 10.19 d). The negative impact of heat on conception rates (Lucy, 2002), on number of days open (El-Tarabany and El-Tarabany, 2015), and on nonreturn rates (Ravagnolo and Misztal, 2002) is possibly related to hormone secretions of cows, influenced by high ambient temperatures. For example, HS reduced plasma concentrations of estradiol (Wolfenson et al., 1995) or progesterone and increased plasma concentrations of prolactin (Ronchi et al., 2001). A reduced estradiol secretion contributed to a poor expression of estrus and ovulatory failure (De Rensis and Scaramuzzi, 2003), and to the prevention of luteolysis of the corpus luteum (Lucy, 2002). Furthermore, heat compromised size (Schüller et al. 2017), growth (Wilson et al., 1998), and composition of the fluid (Alves et al., 2014) of the ovarian follicle. Due to carryover effects from summer, HS-induced oocyte damage also impairs fertility in autumn. Hence, reproductive performance remains low for approximately 2 mo of cooler temperatures in autumn, when compared with winter (Negrón-Pérez et al., 2019). Furthermore, the early embryo is very sensitive to HS, because high ambient temperatures on day 1 after insemination decreased subsequent embryonic development (Ealy et al., 1993), possibly contributing to embryonic losses and prolonged calving intervals.

**Fig. 6.**
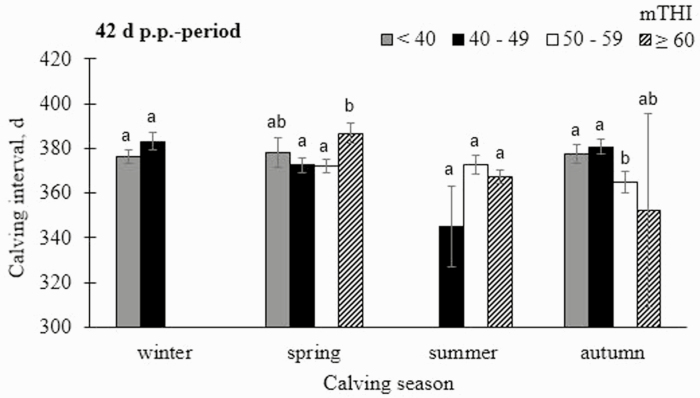
Least-squares means (LSMeans) with corresponding SE for calving interval (CINT) in dependency of mean daily temperature humidity index (mTHI), nested within calving season from the postnatal 42 d-period. Significances of pairwise comparisons are indicated with different letters (a and b) (*P* < 0.05).

The fixed effect of nested mTHI-class within calving season was significant across all a.p.- and p.p.-periods (*P* < 0.001) (model 1). CINT was extended for high mTHI (from 50) before spring-calvings across all a.p.-periods (e.g. 391.6 ± 3.82 d [56 d-period]) (Supplementary Table S2). With regard to p.p.-periods, longest CINT were found when mTHI was ≥ 60 after spring-calvings (e.g. 386.8 ± 4.62 d [42 d p.p.-period]; Fig. 6). The fixed effect of nested nHS within calving season was significant for all a.p.-periods (7 d-period: *P* < 0.001; 42 d-period: *P* < 0.05; 56 d-period: *P* < 0.05) and p.p.-periods (*P* < 0.001) (model 2). A number of 6 to 7 HS days during the 7 d-period before autumn calvings caused an exceptionally long CINT of 401.2 d (± 15.45 d). It was mentioned previously that cows can adapt to climatic conditions by gradual acclimatization (Prosser, 2013). It allows cows to cope with heat loads during the hot summer months and refers to short-term and long-term acclimatization processes. The short-term heat acclimatization phase initiates cellular changes (Horowitz, 2001) with regard to compensate acute increased HS conditions. When HS conditions remain constant, it is followed by the long-term heat acclimatization phase (Bernabucci et al., 2010). This phase includes reprogramming of gene expressions and cellular responses with positive effect on metabolic processes (Horowitz, 2002), and endocrine changes contributing to metabolic heat production (Bernabucci et al. 2010). In autumn, HS appears less frequently than in summer, why acclimatization of cows to heat corresponds to short-term acclimatization processes, only. The high nHS in the week before autumn-calvings resulted in an exceptionally long CINT. Interestingly, we found tendencies for shortest CINT in cows with a calving date under HS conditions during the summer months. Accordingly, Tao et al. (2012) identified shortened gestation periods for cows that were heat stressed during the dry period. In our study, cows calving during the summer months calved again in the summer season of the following year, implying direct HS around the calving date 1 yr later. In addition, explanations for shortened CINT address time-lagged HS-effects, leading to shortened lengths of the following gestation period. With regard to the p.p.-periods, longest CINT were found for high nHS after spring-calvings (e.g., 3 to 5 HS days in the 7 d-period: 390.7 ± 5.83 d or 31 to 40 HS days in the 56 d-period: 389.7 ± 4.90 d) (Supplementary Table S2). Mathevon et al. (1998) found a significantly reduced semen concentration and a significantly lower number of total and motile sperms in bulls during summer, when compared with winter and spring. Furthermore, Nichi et al. (2006) described a tendency to greater total sperm defects in bulls during the summer season. Combined with a lower level of acclimatization to heat of cows in spring, a heat-induced reduction of semen quality also contributes to lower conception rates in cows, explaining prolonged CINT after spring-calvings.

In the present study, detrimental impact of HS from different a.p.- and p.p.-periods on production and fertility traits of an endangered beef cattle breed kept under pasture-based conditions was proven. The strongest detrimental effect on BWT of calves was found for high mTHI or high nHS before autumn-births. This result indicates an increasing sensitivity to HS during the generally cooler autumn months, compared with hot summer months. Similar HS influence was detected for SB, with increasing probabilities due to increasing mTHI and nHS. Impairing effects of heat on weight gains of calves were most obvious when evaluating HS indicators during the long-term postnatal periods (42 d- and 56 d-periods). Furthermore, calves born in summer who suffered high mTHI or high nHS pre- or postnatal had lower weight gains compared with calves born in other calving seasons or under cooler conditions. Prolonged CINT especially appeared when cows suffered HS after spring calvings, reflecting the high sensitivity of cows to heat during the generally cooler months.

The present study underlines the importance of the HS topic regarding production and functional trait responses, also for “robust” local cattle breeds. Results indicate acute and time-lagged HS-effects and address possible HS-induced epigenetic modifications of the bovine genome across generations and limited acclimatization processes to heat, especially when heat occurs during the cooler spring and autumn months. In pasture-based production systems, commercial cooling systems, which are commonly used in dairy cattle sheds (fans and sprinklers), cannot be deployed. Provision of shade, however, can improve performance traits of heat-stressed feedlot cattle (Mitlöhner et al., 2001). In this regard, we highly recommend improving grazing conditions, also by guaranteeing unlimited access to water, especially when heat lasts for several weeks or when temperatures are unexpectedly high during the cooler spring or autumn months. In addition, we suggest a further enhancement of robustness via optimized breeding strategies and of deeper investigations to infer causalities for time-lagged HS influences.

## Supplementary Material

txaa148_suppl_Supplementary_Table_S1

txaa148_suppl_Supplementary_Table_S2

txaa148_suppl_Supplementary_Table_S3
