# Supplementary material for: Time-lagged and acute impact of heat stress on production and fertility traits in the local dual-purpose cattle breed “Rotes Höhenvieh” under pasture-based conditions
Source: Transl Anim Sci. 2020 Aug 5;4(3):txaa148. doi: 10.1093/tas/txaa148 (PMC7528550; doi:10.1093/tas/txaa148)
Supplement: txaa148_suppl_Supplementary_Table_S1 [file txaa148_suppl_supplementary_table_s1.docx]

**Supplemental Material**

**Table S1:** Effects of mTHI-class or nHS-class during the different recording periods for HS indicators on production and fertility traits. The table includes Least-squares means (LSMeans)

with corresponding SE, number of observations (n) and P-value.

| Recording period for HS indicators | Fixed effect | Trait^1^ | | | | | | | | | | | | | | | |
| --- | --- | --- | --- | --- | --- | --- | --- | --- | --- | --- | --- | --- | --- | --- | --- | --- | --- |
|  |  | BWT, kg | | |  | 200dg, kg | | |  | 365dg, kg | | |  | CINT, d | | | |
|  |  | LSMeans^4^ | SE^5^ | *n*^6^ | *P*-value | LSMeans | SE | *n* | *P*-value | LSMeans | SE | *n* | *P*-value | LSMeans | SE | *n* | *P*-value |
|  | mTHI^2^-class |  | | | > 0.05 |  | | | < 0.05 |  | | | > 0.05 |  | | | > 0.05 |
| 7 d prepartum / a.p. | < 40 | 36.7 | 0.2357 | 1,264 |  | 209.3 | 3.2781 | 760 |  | 331.3 | 4.1390 | 706 |  | 374.9 | 3.0028 | 820 |  |
|  | 40 - 49 | 36.6 | 0.3001 | 1,061 |  | 218.5 | 4.3943 | 748 |  | 326.3 | 5.3379 | 623 |  | 370.6 | 3.8611 | 643 |  |
|  | 50 - 59 | 36.6 | 1.0450 | 1,218 |  | 208.3 | 2.9359 | 942 |  | 319.7 | 13.2463 | 725 |  | 371.9 | 11.1657 | 855 |  |
|  | ≥ 60 | 36.8 | 0.2757 | 819 |  | 202.4 | 3.7588 | 686 |  | 335.5 | 4.5842 | 448 |  | 375.4 | 3.7371 | 487 |  |
|  | nHS^3^-class |  | | | < 0.05 |  | | | < 0.01 |  | | | < 0.01 |  | | | > 0.05 |
| 7 d prepartum / a.p. | 1 | 36.7 | 0.1828 | 3,439 |  | 210.8 | 2.6089 | 2,368 |  | 327.5 | 2.9126 | 1,988 |  | 376.2 | 2.2276 | 2,254 |  |
|  | 2 | 37.1 | 0.2807 | 631 |  | 209.2 | 3.8466 | 512 |  | 340.3 | 4.5357 | 365 |  | 377.6 | 3.7317 | 373 |  |
|  | 3 | 35.8 | 0.6108 | 292 |  | 179.1 | 10.5243 | 256 |  | 320.3 | 10.8984 | 149 |  | 371.5 | 8.2841 | 178 |  |
|  | mTHI-class |  | | | > 0.05 |  | | | > 0.05 |  | | | < 0.05 |  | | | > 0.05 |
| 42 d prepartum / a.p. | < 40 | 36.5 | 0.4774 | 1,325 |  | 205.8 | 6.8362 | 787 |  | 332.6 | 11.6800 | 734 |  | 370.6 | 4.7179 | 867 |  |
|  | 40 - 49 | 36.7 | 0.2897 | 1,109 |  | 210.3 | 4.2056 | 794 |  | 324.5 | 5.2944 | 665 |  | 373.2 | 4.1258 | 676 |  |
|  | 50 - 59 | 37.1 | 0.7480 | 1,329 |  | 189.3 | 9.0785 | 1,035 |  | 349.8 | 9.4944 | 817 |  | 394.9 | 11.0563 | 873 |  |
|  | ≥ 60 | 36.2 | 0.2642 | 599 |  | 201.7 | 3.5834 | 520 |  | 338.3 | 4.5732 | 286 |  | 368.4 | 3.3778 | 388 |  |
|  | nHS-class |  | | | > 0.05 |  | | | < 0.05 |  | | | < 0.001 |  | | | < 0.01 |
| 42 d prepartum / a.p. | 1 | 36.7 | 0.1881 | 3,275 |  | 207.8 | 2.6632 | 2,276 |  | 329.4 | 3.0140 | 1,925 |  | 376.5 | 2.3367 | 2,092 |  |
|  | 2 | 36.8 | 0.3399 | 480 |  | 211.3 | 6.1910 | 327 |  | 315.4 | 5.1666 | 289 |  | 381.0 | 4.2998 | 314 |  |
|  | 3 | 36.5 | 1.4227 | 379 |  | 185.3 | 17.2379 | 336 |  | 341.3 | 5.4769 | 164 |  | 369.5 | 3.7446 | 260 |  |
|  | 4 | 36.7 | 0.4039 | 228 |  | 195.6 | 5.2391 | 197 |  | 338.4 | 6.1778 | 124 |  | 362.0 | 5.1608 | 138 |  |
|  | mTHI-class |  | | | > 0.05 |  | | | > 0.05 |  | | | > 0.05 |  | | | > 0.05 |
| 56 d prepartum / a.p. | < 40 | 36.3 | 0.6510 | 1,342 |  | 209.9 | 9.9807 | 787 |  | 327.3 | 13.3717 | 740 |  | 367.3 | 7.5889 | 875 |  |
|  | 40 - 49 | 36.7 | 0.2629 | 1,125 |  | 204.9 | 3.6658 | 835 |  | 326.3 | 5.0229 | 666 |  | 373.5 | 3.6541 | 699 |  |
|  | 50 - 59 | 37.1 | 0.5500 | 1,322 |  | 189.2 | 9.0742 | 1,022 |  | 338.6 | 7.1459 | 803 |  | 384.3 | 8.0176 | 856 |  |
|  | ≥ 60 | 36.3 | 0.2533 | 573 |  | 200.0 | 3.4885 | 492 |  | 338.4 | 4.2219 | 293 |  | 366.1 | 3.1975 | 379 |  |
|  |  |  |  |  |  |  |  |  |  |  |  |  |  |  |  |  |  |
|  | nHS-class |  | | | > 0.05 |  | | | > 0.05 |  | | | < 0.01 |  | | | < 0.001 |
| 56 d prepartum / a.p. | 1 | 36.7 | 0.1930 | 3,098 |  | 207.3 | 2.7299 | 2,146 |  | 327.7 | 3.1317 | 1,825 |  | 378.9 | 2.3905 | 1,976 |  |
|  | 2 | 36.9 | 0.3374 | 469 |  | 210.3 | 6.1795 | 322 |  | 318.6 | 5.1416 | 285 |  | 380.2 | 4.2658 | 319 |  |
|  | 3 | 37.2 | 1.4223 | 325 |  | 184.6 | 17.2755 | 254 |  | 333.7 | 5.0880 | 148 |  | 373.1 | 3.7310 | 217 |  |
|  | 4 | 36.1 | 0.3210 | 274 |  | 206.5 | 4.2332 | 243 |  | 338.6 | 5.4760 | 141 |  | 366.4 | 4.1072 | 172 |  |
|  | 5 | 36.1 | 0.3691 | 196 |  | 201.1 | 4.8846 | 171 |  | 342.2 | 6.0825 | 103 |  | 360.8 | 4.8103 | 125 |  |
|  | mTHI-class |  | | |  |  | | | > 0.05 |  | | | < 0.05 |  | | | > 0.05 |
| 7 d postpartum / p.p. | < 40 |  |  |  |  | 209.3 | 3.1472 | 743 |  | 333.6 | 3.8336 | 669 |  | 379.2 | 2.8827 | 774 |  |
|  | 40 - 49 |  |  |  |  | 211.4 | 5.0152 | 715 |  | 344.8 | 6.2003 | 619 |  | 371.3 | 4.8111 | 665 |  |
|  | 50 - 59 |  |  |  |  | 210.5 | 12.4718 | 960 |  | 314.8 | 9.5379 | 733 |  | 379.0 | 6.7791 | 848 |  |
|  | ≥ 60 |  |  |  |  | 203.9 | 4.4086 | 717 |  | 342.6 | 5.5114 | 481 |  | 373.7 | 4.1928 | 510 |  |
|  | nHS-class |  | | |  |  | | | > 0.05 |  | | | < 0.01 |  | | | > 0.05 |
| 7 d postpartum / p.p. | 1 |  |  |  |  | 211.0 | 2.5991 | 2,349 |  | 328.1 | 2.9133 | 1,966 |  | 375.2 | 2.2530 | 2,236 |  |
|  | 2 |  |  |  |  | 204.9 | 4.2083 | 570 |  | 340.9 | 4.8498 | 398 |  | 373.2 | 3.7904 | 390 |  |
|  | 3 |  |  |  |  | 202.0 | 6.8506 | 216 |  | 329.2 | 10.6190 | 138 |  | 381.9 | 7.6316 | 171 |  |
|  | mTHI-class |  | | |  |  | | | > 0.05 |  | | | < 0.05 |  | | | > 0.05 |
| 42 d postpartum / p.p. | < 40 |  |  |  |  | 211.0 | 3.5767 | 713 |  | 334.1 | 4.7237 | 619 |  | 377.2 | 3.2161 | 708 |  |
|  | 40 - 49 |  |  |  |  | 216.8 | 4.9642 | 757 |  | 343.3 | 7.1903 | 652 |  | 370.3 | 5.0488 | 754 |  |
|  | 50 - 59 |  |  |  |  | 213.5 | 2.9944 | 852 |  | 332.1 | 3.6270 | 629 |  | 369.9 | 2.8512 | 736 |  |
|  | ≥ 60 |  |  |  |  | 229.3 | 16.3084 | 814 |  | 323.8 | 3.8180 | 602 |  | 368.7 | 14.7428 | 600 |  |
|  | nHS-class |  | | |  |  | | | < 0.001 |  | | | < 0.001 |  | | | > 0.05 |
| 42 d postpartum / p.p. | 1 |  |  |  |  | 213.9 | 2.7923 | 1,959 |  | 333.4 | 3.3459 | 1,606 |  | 375.4 | 2.4776 | 1,899 |  |
|  | 2 |  |  |  |  | 199.3 | 5.8044 | 400 |  | 340.7 | 6.9775 | 328 |  | 377.2 | 5.6385 | 347 |  |
|  | 3 |  |  |  |  | 229.5 | 16.5928 | 408 |  | 318.8 | 4.3763 | 310 |  | 372.0 | 14.7101 | 359 |  |
|  | 4 |  |  |  |  | 194.2 | 4.1516 | 369 |  | 333.5 | 4.8790 | 258 |  | 398.0 | 10.1892 | 193 |  |
|  | mTHI-class |  | | |  |  | | | < 0.001 |  | | | < 0.01 |  | | | > 0.05 |
| 56 d postpartum / p.p. | < 40 |  |  |  |  | 212.3 | 3.8633 | 716 |  | 332.4 | 5.5634 | 611 |  | 377.9 | 3.6670 | 687 |  |
|  | 40 - 49 |  |  |  |  | 219.2 | 4.4852 | 706 |  | 342.2 | 6.2032 | 615 |  | 376.1 | 4.1678 | 737 |  |
|  | 50 - 59 |  |  |  |  | 212.2 | 3.0832 | 839 |  | 334.0 | 3.7748 | 597 |  | 374.0 | 15.2317 | 736 |  |
|  | ≥ 60 |  |  |  |  | 201.6 | 3.1867 | 875 |  | 324.1 | 3.7148 | 679 |  | 380.6 | 2.9976 | 651 |  |
|  |  |  |  |  |  |  |  |  |  |  |  |  |  |  |  |  |  |
|  | nHS-class |  | | |  |  | | | < 0.001 |  | | | < 0.001 |  | | | > 0.05 |
| 56 d postpartum / p.p. | 1 |  |  |  |  | 216.3 | 2.8826 | 1,800 |  | 337.6 | 3.5404 | 1,485 |  | 377.5 | 2.5505 | 1,739 |  |
|  | 2 |  |  |  |  | 198.3 | 6.0344 | 299 |  | 336.7 | 7.3635 | 221 |  | 372.0 | 5.9829 | 268 |  |
|  | 3 |  |  |  |  | 218.1 | 11.9836 | 274 |  | 356.7 | 17.2388 | 235 |  | 364.6 | 10.6006 | 281 |  |
|  | 4 |  |  |  |  | 205.7 | 3.6584 | 415 |  | 317.2 | 4.2940 | 316 |  | 381.1 | 3.5226 | 334 |  |
|  | 5 |  |  |  |  | 196.3 | 4.0692 | 348 |  | 335.8 | 4.7560 | 245 |  | 371.5 | 13.1054 | 189 |  |

^1^Traits: BWT = birth weight; 200dg = 200 d-weight gain; 365dg = 365 d-weight gain; CINT = calving interval.

^2^mTHI = mean daily temperature humidity index.

^3^nHS = number of heat stress days.

^4^LSMeans = Least-squares means.

^5^SE = Standard error.

^6^n = number of observations.
