# Supplementary material for: Time-lagged and acute impact of heat stress on production and fertility traits in the local dual-purpose cattle breed “Rotes Höhenvieh” under pasture-based conditions
Source: Transl Anim Sci. 2020 Aug 5;4(3):txaa148. doi: 10.1093/tas/txaa148 (PMC7528550; doi:10.1093/tas/txaa148)
Supplement: txaa148_suppl_Supplementary_Table_S2 [file txaa148_suppl_supplementary_table_s2.docx]

**Supplememtal Metrial**

**Table S2:** Effects of nested mTHI-class or nested nHS-class within calving season during the different recording periods on production and fertility traits. The table includes

Least-squares means (LSMeans) with corresponding SE, number of observations (n) and P-value.

| Trait^1^ | Recording period for HS indicators | Nested effect | Calving season | | | | | | | | | | | | *P*-value |
| --- | --- | --- | --- | --- | --- | --- | --- | --- | --- | --- | --- | --- | --- | --- | --- |
|  |  |  | Winter | | | Spring | | | Summer | | | Autumn | | |  |
|  |  |  | LSMeans^4^ | SE^5^ | *n*^6^ | LSMeans | SE | *n* | LSMeans | SE | *n* | LSMeans | SE | *n* |  |
| BW, kg |  | mTHI^2^-class |  | | | | | | | | | | | | < 0.01 |
|  | 7 d prepartum / a.p. | < 40 | 36.7 | 0.2203 | 836 | 36.8 | 0.2915 | 322 |  |  |  | 36.6 | 0.4424 | 106 |  |
|  |  | 40 - 49 | 36.6 | 0.3638 | 171 | 36.4 | 0.2428 | 570 | 37.1 | 0.9442 | 21 | 36.5 | 0.2938 | 299 |  |
|  |  | 50 - 59 | 36.1 | 4.1091 | 1 | 36.7 | 0.2522 | 521 | 37.1 | 0.2784 | 371 | 36.5 | 0.2861 | 325 |  |
|  |  | ≥ 60 |  |  |  | 37.9 | 0.4328 | 114 | 37.2 | 0.2450 | 624 | 35.3 | 0.4957 | 81 |  |
|  |  | nHS^3^-class |  | | | | | | | | | | | | < 0.01 |
|  | 7 d prepartum / a.p. | 1 | 36.7 | 0.2103 | 1,008 | 36.6 | 0.2009 | 1,365 | 37.1 | 0.2841 | 349 | 36.5 | 0.2286 | 717 |  |
|  |  | 2 |  |  |  | 37.8 | 0.3827 | 155 | 37.4 | 0.2687 | 411 | 36.2 | 0.5466 | 65 |  |
|  |  | 3 |  |  |  | 36.1 | 1.5657 | 7 | 36.9 | 0.3178 | 256 | 34.4 | 0.7931 | 29 |  |
|  |  | mTHI-class |  | | | | | | | | | | | | < 0.001 |
|  | 42 d prepartum / a.p. | < 40 | 36.6 | 0.2226 | 813 | 36.7 | 0.2562 | 502 |  |  |  | 36.1 | 1.3295 | 10 |  |
|  |  | 40 - 49 | 36.8 | 0.3462 | 193 | 36.3 | 0.2355 | 608 | 37.0 | 0.8937 | 24 | 36.5 | 0.3068 | 284 |  |
|  |  | 50 - 59 | 37.5 | 2.8902 | 2 | 37.2 | 0.2781 | 417 | 37.1 | 0.2512 | 543 | 36.8 | 0.2725 | 367 |  |
|  |  | ≥ 60 |  |  |  |  |  |  | 37.3 | 0.2678 | 449 | 35.2 | 0.3844 | 150 |  |
|  |  | nHS-class |  | | | | | | | | | | | | < 0.001 |
|  | 42 d prepartum / a.p. | 1 | 36.7 | 0.2103 | 1,008 | 36.6 | 0.1987 | 1,496 | 36.9 | 0.3261 | 234 | 36.7 | 0.2474 | 537 |  |
|  |  | 2 |  |  |  | 36.9 | 0.7785 | 30 | 37.1 | 0.2903 | 325 | 36.3 | 0.4084 | 125 |  |
|  |  | 3 |  |  |  | 37.6 | 4.2153 | 1 | 37.3 | 0.3114 | 269 | 34.7 | 0.4351 | 109 |  |
|  |  | 4 |  |  |  |  |  |  | 37.3 | 0.3543 | 188 | 36.1 | 0.6815 | 40 |  |
|  |  |  |  |  |  |  |  |  |  |  |  |  |  |  |  |
|  |  | mTHI-class |  | | | | | | | | | | | | < 0.001 |
|  | 56 d prepartum / a.p. | < 40 | 36.8 | 0.227 | 754 | 36.8 | 0.2456 | 583 |  |  |  | 35.3 | 1.8854 | 5 |  |
|  |  | 40 - 49 | 36.5 | 0.316 | 250 | 36.3 | 0.2368 | 603 | 37.4 | 0.7462 | 35 | 36.7 | 0.3267 | 237 |  |
|  |  | 50 - 59 | 37.3 | 2.0576 | 4 | 37.3 | 0.2985 | 341 | 37.2 | 0.2449 | 613 | 36.8 | 0.2743 | 364 |  |
|  |  | ≥ 60 |  |  |  |  |  |  | 37.2 | 0.2834 | 368 | 35.4 | 0.3412 | 205 |  |
|  |  | nHS-class |  | | | | | | | | | | | | < 0.001 |
|  | 56 d prepartum / a.p. | 1 | 36.7 | 0.2104 | 1,008 | 36.7 | 0.1987 | 1,496 | 36.7 | 0.3525 | 188 | 36.7 | 0.2703 | 406 |  |
|  |  | 2 |  |  |  | 36.9 | 0.778 | 30 | 37.4 | 0.2972 | 302 | 36.4 | 0.3933 | 137 |  |
|  |  | 3 |  |  |  | 37.6 | 4.2124 | 1 | 37.1 | 0.3418 | 204 | 37.0 | 0.4172 | 120 |  |
|  |  | 4 |  |  |  |  |  |  | 37.6 | 0.357 | 185 | 34.7 | 0.4742 | 89 |  |
|  |  | 5 |  |  |  |  |  |  | 36.5 | 0.398 | 137 | 35.6 | 0.5744 | 59 |  |
| 200dg, kg |  | mTHI-class |  |  |  |  |  |  |  |  |  |  |  |  | < 0.001 |
|  | 7 d prepartum / a.p. | < 40 | 197.3 | 3.3336 | 455 | 240.1 | 4.1953 | 216 |  |  |  | 190.6 | 5.9384 | 89 |  |
|  |  | 40 - 49 | 214.5 | 5.4127 | 109 | 230.1 | 3.4921 | 390 | 224.0 | 14.1970 | 14 | 205.4 | 4.1558 | 235 |  |
|  |  | 50 - 59 |  |  |  | 214.6 | 3.5174 | 412 | 197.6 | 3.9471 | 264 | 212.7 | 3.9331 | 266 |  |
|  |  | ≥ 60 |  |  |  | 208.9 | 5.8814 | 90 | 192.4 | 3.3841 | 526 | 205.9 | 6.4783 | 70 |  |
|  |  | nHS-class |  | | | | | | | | | | | | < 0.001 |
|  | 7 d prepartum / a.p. | 1 | 201.9 | 3.1517 | 564 | 227.9 | 2.8463 | 973 | 205.6 | 4.0265 | 251 | 207.9 | 3.2781 | 580 |  |
|  |  | 2 |  |  |  | 211.9 | 5.0899 | 132 | 194.8 | 3.7290 | 327 | 221.0 | 7.3426 | 53 |  |
|  |  | 3 |  |  |  | 157.7 | 29.1025 | 3 | 189.4 | 4.2393 | 226 | 190.2 | 10.0179 | 27 |  |
|  |  | mTHI-class |  | | | | | | | | | | | | < 0.001 |
|  | 42 d prepartum / a.p. | < 40 | 202.7 | 3.3617 | 438 | 240.4 | 3.6471 | 342 |  |  |  | 174.3 | 19.0285 | 7 |  |
|  |  | 40 - 49 | 194.6 | 5.1852 | 124 | 223.7 | 3.409 | 419 | 222.7 | 13.2178 | 16 | 200.9 | 4.2557 | 235 |  |
|  |  | 50 - 59 | 141.2 | 34.6205 | 2 | 211.0 | 3.8092 | 347 | 198.6 | 3.5246 | 403 | 206.5 | 3.8639 | 283 |  |
|  |  | ≥ 60 |  |  |  |  |  |  | 187.8 | 3.6571 | 385 | 215.5 | 5.0119 | 135 |  |
|  |  |  |  |  |  |  |  |  |  |  |  |  |  |  |  |
|  |  | nHS-class |  | | | | | | | | | | | | < 0.001 |
|  | 42 d prepartum / a.p. | 1 | 201.8 | 3.1499 | 564 | 226.2 | 2.8079 | 1,097 | 198.3 | 4.4261 | 189 | 204.7 | 3.547 | 426 |  |
|  |  | 2 |  |  |  | 217.2 | 16.1797 | 10 | 206.5 | 4.191 | 218 | 210.2 | 5.612 | 99 |  |
|  |  | 3 |  |  |  | 133.1 | 50.9272 | 1 | 199.6 | 4.1601 | 235 | 223.2 | 5.5931 | 100 |  |
|  |  | 4 |  |  |  |  |  |  | 180.9 | 4.7317 | 162 | 210.4 | 6.783 | 35 |  |
|  |  | mTHI-class |  | | | | | | | | | | | | < 0.001 |
|  | 56 d prepartum / a.p. | < 40 | 204.3 | 3.4641 | 396 | 238.1 | 3.5045 | 388 |  |  |  | 187.1 | 29.032 | 3 |  |
|  |  | 40 - 49 | 191.5 | 4.6538 | 166 | 222.0 | 3.378 | 438 | 211.6 | 10.3295 | 27 | 194.4 | 4.4666 | 204 |  |
|  |  | 50 - 59 | 140.9 | 34.5565 | 2 | 209.5 | 4.0697 | 282 | 199.0 | 3.4177 | 462 | 207.4 | 3.8928 | 276 |  |
|  |  | ≥ 60 |  |  |  |  |  |  | 184.6 | 3.854 | 315 | 215.3 | 4.5583 | 177 |  |
|  |  | nHS-class |  | | | | | | | | | | | | < 0.001 |
|  | 56 d prepartum / a.p. | 1 | 201.7 | 3.1548 | 564 | 226.5 | 2.8096 | 1,097 | 199.2 | 4.7937 | 152 | 202.0 | 3.8339 | 333 |  |
|  |  | 2 |  |  |  | 217.6 | 16.1842 | 10 | 199.3 | 4.2488 | 212 | 214.0 | 5.6325 | 100 |  |
|  |  | 3 |  |  |  | 133.9 | 50.9403 | 1 | 210.4 | 4.6919 | 161 | 209.4 | 5.8545 | 92 |  |
|  |  | 4 |  |  |  |  |  |  | 191.8 | 4.75 | 161 | 221.3 | 6.0581 | 82 |  |
|  |  | 5 |  |  |  |  |  |  | 183.2 | 5.2935 | 118 | 219.1 | 7.3812 | 53 |  |
|  |  | mTHI-class |  | | | | | | | | | | | | < 0.001 |
|  | 7 d postpartum / p.p. | < 40 | 196.9 | 3.3423 | 444 | 239.3 | 4.582 | 164 |  |  |  | 191.8 | 5.0737 | 135 |  |
|  |  | 40 - 49 | 213.2 | 5.1629 | 119 | 233.7 | 3.6019 | 346 | 195.5 | 16.9047 | 9 | 203.2 | 4.0553 | 241 |  |
|  |  | 50 - 59 | 204.3 | 3.9743 | 1 | 215.4 | 3.3951 | 455 | 206.3 | 3.9743 | 247 | 216.1 | 3.9806 | 257 |  |
|  |  | ≥ 60 |  |  |  | 211.7 | 4.9633 | 142 | 189.0 | 3.3692 | 548 | 211.1 | 9.8042 | 27 |  |
|  |  | nHS-class |  | | | | | | | | | | | | < 0.001 |
|  | 7 d postpartum / p.p. | 1 | 201.7 | 3.1551 | 564 | 227.6 | 2.8658 | 922 | 206.7 | 4.0265 | 246 | 208.1 | 3.2438 | 617 |  |
|  |  | 2 |  |  |  | 215.3 | 4.7153 | 165 | 195.1 | 3.656 | 373 | 204.3 | 9.1511 | 32 |  |
|  |  | 3 |  |  |  | 203.5 | 11.4295 | 20 | 184.7 | 4.5016 | 185 | 217.9 | 15.1247 | 11 |  |
|  |  |  |  |  |  |  |  |  |  |  |  |  |  |  |  |
|  |  |  |  |  |  |  |  |  |  |  |  |  |  |  |  |
|  |  | mTHI-class |  | | | | | | | | | | | | < 0.001 |
|  | 42 d postpartum / p.p. | < 40 | 188.5 | 3.3587 | 422 | 246.8 | 7.327 | 54 |  |  |  | 197.6 | 4.1669 | 237 |  |
|  |  | 40 - 49 | 236.1 | 4.7627 | 142 | 237.8 | 3.5582 | 335 | 184.6 | 16.9331 | 10 | 208.9 | 3.8538 | 270 |  |
|  |  | 50 - 59 |  |  |  | 221.6 | 3.2905 | 471 | 203.2 | 4.107 | 229 | 215.6 | 4.7123 | 152 |  |
|  |  | ≥ 60 |  |  |  | 212.5 | 4.1328 | 248 | 192.1 | 3.3476 | 565 | 283.2 | 48.1902 | 1 |  |
|  |  | nHS-class |  | | | | | | | | | | | | < 0.001 |
|  | 42 d postpartum / p.p. | 1 | 201.6 | 3.1389 | 564 | 233.7 | 3.0648 | 639 | 212.7 | 5.5864 | 108 | 207.4 | 3.2004 | 648 |  |
|  |  | 2 |  |  |  | 214.2 | 4.0834 | 253 | 198.5 | 4.9298 | 136 | 185.2 | 15.0528 | 11 |  |
|  |  | 3 |  |  |  | 211.7 | 5.0743 | 135 | 197.3 | 4.0231 | 272 | 279.7 | 48.9151 | 1 |  |
|  |  | 4 |  |  |  | 205.8 | 6.1676 | 84 | 182.5 | 3.9843 | 288 |  |  |  |  |
|  |  | mTHI-class |  | | | | | | | | | | | | < 0.001 |
|  | 56 d postpartum / p.p. | < 40 | 187.4 | 3.4236 | 406 | 248.5 | 8.5154 | 39 |  |  |  | 201.1 | 3.1867 | 271 |  |
|  |  | 40 - 49 | 233.1 | 4.5963 | 158 | 238.6 | 3.9375 | 253 | 196.8 | 14.6602 | 13 | 208.1 | 3.8151 | 282 |  |
|  |  | 50 - 59 |  |  |  | 224.2 | 3.2852 | 471 | 201.8 | 3.9323 | 261 | 210.5 | 5.4131 | 107 |  |
|  |  | ≥ 60 |  |  |  | 212.7 | 3.8132 | 345 | 190.5 | 3.4299 | 530 |  |  |  |  |
|  |  | nHS-class |  | | | | | | | | | | | | < 0.001 |
|  | 56 d postpartum / p.p. | 1 | 202.0 | 3.1349 | 564 | 237.6 | 3.2614 | 502 | 217.5 | 6.1047 | 86 | 208.0 | 3.199 | 648 |  |
|  |  | 2 |  |  |  | 217.4 | 4.4856 | 174 | 194.4 | 5.3297 | 115 | 183.1 | 15.7526 | 10 |  |
|  |  | 3 |  |  |  | 214.3 | 4.6182 | 172 | 193.0 | 5.6255 | 100 | 247.1 | 34.6724 | 2 |  |
|  |  | 4 |  |  |  | 213.6 | 4.7244 | 166 | 197.7 | 4.15 | 249 |  |  |  |  |
|  |  | 5 |  |  |  | 208.0 | 5.8273 | 94 | 184.5 | 4.1191 | 254 |  |  |  |  |
| 365dg, kg |  | mTHI-class |  | | | | | | | | | | | | < 0.001 |
|  | 7 d prepartum / a.p. | < 40 | 325.8 | 3.4908 | 512 | 336.8 | 5.0318 | 155 |  |  |  | 331.3 | 8.8672 | 39 |  |
|  |  | 40 - 49 | 318.0 | 5.4368 | 126 | 333.1 | 3.9300 | 327 | 308.6 | 17.8605 | 9 | 345.5 | 4.8613 | 161 |  |
|  |  | 50 - 59 | 293.1 | 51.5756 | 1 | 329.0 | 3.9780 | 349 | 312.9 | 4.6318 | 200 | 343.8 | 4.7382 | 175 |  |
|  |  | ≥ 60 |  |  |  | 331.7 | 6.3856 | 86 | 322.9 | 4.0543 | 324 | 352.0 | 8.9578 | 38 |  |
|  |  |  |  |  |  |  |  |  |  |  |  |  |  |  |  |
|  |  | nHS-class |  | | | | | | | | | | | | < 0.001 |
|  | 7 d prepartum / a.p. | 1 | 324.3 | 3.3121 | 639 | 332.7 | 3.2200 | 795 | 310.2 | 4.7455 | 185 | 343.0 | 3.7251 | 369 |  |
|  |  | 2 |  |  |  | 330.5 | 5.6526 | 118 | 326.4 | 4.4952 | 211 | 364.0 | 9.1343 | 36 |  |
|  |  | 3 |  |  |  | 318.5 | 25.5540 | 4 | 321.5 | 5.2966 | 137 | 320.9 | 18.3033 | 8 |  |
|  |  | mTHI-class |  | | | | | | | | | | | | < 0.001 |
|  | 42 d prepartum / a.p. | < 40 | 325.3 | 3.558 | 490 | 337.8 | 4.3537 | 241 |  |  |  | 334.6 | 34.3433 | 3 |  |
|  |  | 40 - 49 | 317.1 | 5.056 | 147 | 331.5 | 3.7621 | 372 | 317.5 | 17.7159 | 10 | 331.9 | 5.2859 | 136 |  |
|  |  | 50 - 59 | 410.5 | 35.8978 | 2 | 326.9 | 4.2791 | 304 | 312.7 | 4.1134 | 304 | 349.0 | 4.42 | 207 |  |
|  |  | ≥ 60 |  |  |  |  |  |  | 326.5 | 4.5395 | 219 | 350.1 | 6.949 | 67 |  |
|  |  | nHS-class |  | | | | | | | | | | | | < 0.001 |
|  | 42 d prepartum / a.p. | 1 | 323.6 | 3.2984 | 639 | 332.6 | 3.1701 | 895 | 317.6 | 5.5199 | 117 | 343.6 | 4.0582 | 274 |  |
|  |  | 2 |  |  |  | 297.8 | 11.2951 | 22 | 309.5 | 4.6604 | 195 | 339.0 | 6.6765 | 72 |  |
|  |  | 3 |  |  |  |  |  |  | 320.1 | 5.4074 | 126 | 362.5 | 8.8115 | 38 |  |
|  |  | 4 |  |  |  |  |  |  | 338.3 | 6.0789 | 95 | 338.5 | 10.049 | 29 |  |
|  |  | mTHI-class |  | | | | | | | | | | | | < 0.001 |
|  | 56 d prepartum / a.p. | < 40 | 325.9 | 3.6416 | 447 | 334.0 | 4.1082 | 291 |  |  |  | 322.0 | 39.4488 | 2 |  |
|  |  | 40 - 49 | 317.8 | 4.6728 | 188 | 332.7 | 3.8086 | 363 | 326.9 | 16.337 | 12 | 328.0 | 5.9052 | 103 |  |
|  |  | 50 - 59 | 365.8 | 25.7453 | 4 | 327.4 | 4.5173 | 263 | 313.4 | 4.0343 | 335 | 347.9 | 4.4672 | 201 |  |
|  |  | ≥ 60 |  |  |  |  |  |  | 326.8 | 4.8184 | 186 | 350.0 | 5.7492 | 107 |  |
|  |  | nHS-class |  | | | | | | | | | | | | < 0.001 |
|  | 56 d prepartum / a.p. | 1 | 323.6 | 3.3063 | 639 | 332.5 | 3.1765 | 895 | 315.0 | 6.0609 | 92 | 339.6 | 4.556 | 199 |  |
|  |  | 2 |  |  |  | 297.8 | 11.3117 | 22 | 311.9 | 4.6966 | 188 | 346.1 | 6.5522 | 75 |  |
|  |  | 3 |  |  |  |  |  |  | 320.5 | 6.2794 | 87 | 346.9 | 7.1372 | 61 |  |
|  |  | 4 |  |  |  |  |  |  | 319.4 | 5.971 | 98 | 357.8 | 8.3797 | 43 |  |
|  |  | 5 |  |  |  |  |  |  | 341.2 | 7.0237 | 68 | 343.2 | 9.2007 | 35 |  |
|  |  |  |  |  |  |  |  |  |  |  |  |  |  |  |  |
|  |  |  |  |  |  |  |  |  |  |  |  |  |  |  |  |
|  |  | mTHI-class |  | | | | | | | | | | | | < 0.001 |
|  | 7 d postpartum / p.p. | < 40 | 323.9 | 3.5413 | 487 | 338.4 | 5.5566 | 115 |  |  |  | 338.7 | 7.0857 | 67 |  |
|  |  | 40 - 49 | 327.1 | 5.0015 | 150 | 337.5 | 4.0011 | 295 | 370.5 | 21.9022 | 6 | 344.2 | 4.7547 | 168 |  |
|  |  | 50 - 59 | 280.1 | 36.0686 | 2 | 323.2 | 3.7912 | 401 | 310.3 | 4.8389 | 238 | 345.7 | 4.8516 | 162 |  |
|  |  | ≥ 60 |  |  |  | 346.5 | 5.8976 | 106 | 322.7 | 3.9409 | 124 | 358.6 | 13.1151 | 16 |  |
|  |  | nHS-class |  | | | | | | | | | | | | < 0.001 |
|  | 7 d postpartum / p.p. | 1 | 324.5 | 3.3142 | 639 | 331.7 | 3.2248 | 772 | 312.2 | 4.8363 | 171 | 344.0 | 3.7055 | 384 |  |
|  |  | 2 |  |  |  | 340.2 | 5.4036 | 135 | 323.4 | 4.4196 | 238 | 359.1 | 10.6774 | 25 |  |
|  |  | 3 |  |  |  | 306.0 | 16.4108 | 10 | 324.9 | 5.4301 | 124 | 356.7 | 25.658 | 4 |  |
|  |  | mTHI-class |  | | | | | | | | | | | | < 0.001 |
|  | 42 d postpartum / p.p. | < 40 | 324.7 | 3.5692 | 462 | 344.4 | 11.2815 | 24 |  |  |  | 333.2 | 5.2945 | 133 |  |
|  |  | 40 - 49 | 323.2 | 4.8528 | 177 | 337.6 | 4.1577 | 285 | 359.9 | 26.2155 | 4 | 352.6 | 4.5891 | 186 |  |
|  |  | 50 - 59 |  |  |  | 326.9 | 3.6899 | 416 | 328.4 | 5.5211 | 119 | 341.0 | 5.9997 | 94 |  |
|  |  | ≥ 60 |  |  |  | 332.5 | 4.9054 | 192 | 315.1 | 3.9085 | 410 |  |  |  |  |
|  |  | nHS-class |  | | | | | | | | | | | | < 0.001 |
|  | 42 d postpartum / p.p. | 1 | 324.8 | 3.3088 | 639 | 333.8 | 3.5197 | 515 | 331.0 | 8.1533 | 47 | 343.9 | 3.6667 | 405 |  |
|  |  | 2 |  |  |  | 328.5 | 4.4601 | 240 | 326.9 | 6.4036 | 80 | 366.6 | 18.2688 | 8 |  |
|  |  | 3 |  |  |  | 322.7 | 6.0734 | 99 | 315.0 | 4.6196 | 211 |  |  |  |  |
|  |  | 4 |  |  |  | 349.5 | 7.2255 | 63 | 317.5 | 4.7775 | 195 |  |  |  |  |
|  |  | mTHI-class |  | | | | | | | | | | | | < 0.001 |
|  | 56 d postpartum / p.p. | < 40 | 324.1 | 3.5959 | 443 | 337.6 | 14.5256 | 15 |  |  |  | 335.4 | 5.0478 | 153 |  |
|  |  | 40 - 49 | 324.4 | 4.7499 | 196 | 336.5 | 4.5135 | 219 | 357.1 | 21.9634 | 6 | 351.0 | 4.5921 | 194 |  |
|  |  | 50 - 59 |  |  |  | 327.8 | 3.7436 | 397 | 329.9 | 5.2642 | 134 | 344.5 | 6.9824 | 66 |  |
|  |  | ≥ 60 |  |  |  | 333.6 | 4.4392 | 286 | 314.6 | 3.9925 | 393 |  |  |  |  |
|  |  |  |  |  |  |  |  |  |  |  |  |  |  |  |  |
|  |  |  |  |  |  |  |  |  |  |  |  |  |  |  |  |
|  |  |  |  |  |  |  |  |  |  |  |  |  |  |  |  |
|  |  | nHS-class |  | | | | | | | | | | | | < 0.001 |
|  | 56 d postpartum / p.p. | 1 | 325.3 | 3.2927 | 639 | 337.7 | 3.7514 | 407 | 343.0 | 9.2945 | 34 | 344.3 | 3.6492 | 405 |  |
|  |  | 2 |  |  |  | 320.8 | 5.0036 | 148 | 329.4 | 7.0226 | 66 | 359.7 | 19.4143 | 7 |  |
|  |  | 3 |  |  |  | 327.4 | 4.9115 | 173 | 327.1 | 7.1567 | 61 | 415.5 | 50.5561 | 1 |  |
|  |  | 4 |  |  |  | 323.7 | 5.7173 | 114 | 310.7 | 4.7383 | 202 |  |  |  |  |
|  |  | 5 |  |  |  | 353.6 | 6.7426 | 75 | 318.1 | 4.9555 | 170 |  |  |  |  |
| CINT, d |  | mTHI-class |  | | | | | | | | | | | | < 0.001 |
|  | 7 d a.p. | < 40 | 378.2 | 2..7757 | 546 | 378.9 | 2.7757 | 213 |  |  |  | 380.5 | 5.1733 | 61 |  |
|  |  | 40 - 49 | 379.8 | 4.4055 | 111 | 367.0 | 3.1455 | 339 | 354.5 | 16.8747 | 14 | 383.8 | 3.7429 | 179 |  |
|  |  | 50 - 59 | 397.8 | 25.3816 | 1 | 376.8 | 3.0773 | 360 | 372.4 | 3.6485 | 267 | 368.9 | 3.7468 | 227 |  |
|  |  | ≥ 60 |  |  |  | 387.6 | 6.2234 | 46 | 365.6 | 3.0466 | 384 | 367.9 | 9.1609 | 57 |  |
|  |  | nHS-class |  | | | | | | | | | | | | < 0.001 |
|  | 7 d a.p. | 1 | 378.4 | 2.6083 | 658 | 373.2 | 2.5128 | 887 | 372.3 | 3.6611 | 251 | 376.9 | 2.8989 | 458 |  |
|  |  | 2 |  |  |  | 389.2 | 5.5421 | 67 | 367.4 | 3.3950 | 262 | 363.0 | 7.7831 | 44 |  |
|  |  | 3 |  |  |  | 382.6 | 15.6329 | 4 | 361.8 | 4.1758 | 152 | 401.2 | 15.4472 | 22 |  |
|  |  | mTHI-class |  | | | | | | | | | | | | < 0.001 |
|  | 42 d a.p. | < 40 | 381.1 | 2.7677 | 533 | 375.5 | 3.2512 | 321 |  |  |  | 355.1 | 12.6765 | 13 |  |
|  |  | 40 - 49 | 369.7 | 4.4343 | 127 | 365.7 | 3.072 | 364 | 378.0 | 13.5872 | 11 | 379.4 | 4.0073 | 174 |  |
|  |  | 50 - 59 | 435.8 | 43.1643 | 1 | 389.4 | 3.5652 | 268 | 374.7 | 3.1483 | 370 | 379.8 | 3.5385 | 234 |  |
|  |  | ≥ 60 |  |  |  |  |  |  | 363.0 | 3.4535 | 281 | 373.9 | 4.892 | 107 |  |
|  |  | nHS-class |  | | | | | | | | | | | | < 0.05 |
|  | 42 d a.p. | 1 | 378.8 | 2.6115 | 661 | 374.6 | 2.4948 | 932 | 372.8 | 4.1146 | 163 | 379.9 | 3.2266 | 336 |  |
|  |  | 2 |  |  |  | 396.8 | 9.8362 | 21 | 375.1 | 3.766 | 206 | 371.2 | 5.1716 | 87 |  |
|  |  | 3 |  |  |  |  |  |  | 366.7 | 3.9356 | 186 | 372.2 | 5.6052 | 74 |  |
|  |  | 4 |  |  |  |  |  |  | 352.3 | 4.8578 | 107 | 371.6 | 8.4975 | 31 |  |
|  |  |  |  |  |  |  |  |  |  |  |  |  |  |  |  |
|  |  |  |  |  |  |  |  |  |  |  |  |  |  |  |  |
|  |  | mTHI-class |  | | | | | | | | | | | | < 0.001 |
|  | 56 d a.p. | < 40 | 381.4 | 2.7968 | 503 | 374.1 | 3.1032 | 368 |  |  |  | 346.5 | 22.7756 | 4 |  |
|  |  | 40 - 49 | 369.9 | 4.0615 | 156 | 368.1 | 3.0082 | 380 | 375.7 | 11.2601 | 16 | 380.3 | 4.2225 | 147 |  |
|  |  | 50 - 59 | 392.3 | 30.6282 | 2 | 391.6 | 3.8188 | 211 | 374.8 | 3.0428 | 409 | 378.4 | 3.53 | 234 |  |
|  |  | ≥ 60 |  |  |  |  |  |  | 359.3 | 3.6268 | 234 | 372.3 | 4.3108 | 145 |  |
|  |  | nHS-class |  | | | | | | | | | | | | < 0.05 |
|  | 56 d a.p. | 1 | 378.4 | 2.5998 | 661 | 374.6 | 2.469 | 938 | 373.6 | 4.4752 | 128 | 381.1 | 3.5569 | 249 |  |
|  |  | 2 |  |  |  | 396.3 | 9.8108 | 21 | 374.3 | 3.775 | 206 | 369.8 | 5.0344 | 92 |  |
|  |  | 3 |  |  |  |  |  |  | 370.5 | 4.3783 | 134 | 375.7 | 5.3039 | 83 |  |
|  |  | 4 |  |  |  |  |  |  | 358.4 | 4.7345 | 110 | 374.5 | 6.0443 | 62 |  |
|  |  | 5 |  |  |  |  |  |  | 353.9 | 5.4072 | 81 | 367.6 | 7.1606 | 44 |  |
|  |  | mTHI-class |  | | | | | | | | | | | | < 0.001 |
|  | 7 d p.p. | < 40 | 378.6 | 2.7189 | 526 | 374.4 | 3.71 | 159 |  |  |  | 371.6 | 6.0394 | 89 |  |
|  |  | 40 - 49 | 379.2 | 4.7497 | 128 | 365.5 | 3.1325 | 331 | 358.4 | 12.1416 | 7 | 379.4 | 3.9062 | 199 |  |
|  |  | 50 - 59 | 351.6 | 43.6594 | 3 | 382.1 | 3.1709 | 407 | 375.4 | 3.4483 | 227 | 378.4 | 3.5998 | 211 |  |
|  |  | ≥ 60 |  |  |  | 389.4 | 6.8727 | 59 | 365.4 | 3.157 | 426 | 371.2 | 6.2708 | 25 |  |
|  |  | nHS-class |  | | | | | | | | | | | | < 0.001 |
|  | 7 d p.p. | 1 | 378.2 | 2.6053 | 657 | 373.4 | 2.5071 | 872 | 375.8 | 3.5173 | 228 | 377.1 | 2.9296 | 479 |  |
|  |  | 2 |  |  |  | 390.7 | 5.8311 | 76 | 364.7 | 3.4669 | 278 | 377.4 | 7.0501 | 36 |  |
|  |  | 3 |  |  |  | 388.9 | 21.8437 | 8 | 363.6 | 4.2495 | 154 | 362.0 | 9.8391 | 9 |  |
|  |  | mTHI-class |  | | | | | | | | | | | | < 0.001 |
|  | 42 d p.p. | < 40 | 376.2 | 2.8392 | 494 | 377.9 | 6.6032 | 53 |  |  |  | 377.5 | 4.1565 | 161 |  |
|  |  | 40 - 49 | 383.2 | 4.0237 | 169 | 372.4 | 3.2327 | 320 | 344.9 | 18.1252 | 7 | 380.7 | 3.4512 | 258 |  |
|  |  | 50 - 59 |  |  |  | 372.0 | 2.9711 | 452 | 372.8 | 4.0367 | 179 | 364.7 | 4.8739 | 150 |  |
|  |  | ≥ 60 |  |  |  | 386.8 | 4.6205 | 127 | 367.1 | 3.0849 | 472 | 352.2 | 43.4503 | 1 |  |
|  |  |  |  |  |  |  |  |  |  |  |  |  |  |  |  |
|  |  |  |  |  |  |  |  |  |  |  |  |  |  |  |  |
|  |  | nHS-class |  | | | | | | | | | | | | < 0.001 |
|  | 42 d p.p. | 1 | 378.6 | 2.605 | 663 | 367.4 | 2.7023 | 635 | 377.5 | 5.3668 | 86 | 378.3 | 2.8745 | 515 |  |
|  |  | 2 |  |  |  | 390.1 | 3.6891 | 226 | 36.3 | 4.7587 | 112 | 372.3 | 15.1796 | 9 |  |
|  |  | 3 |  |  |  | 389.2 | 5.3258 | 86 | 370.6 | 3.5382 | 272 | 356.1 | 43.2174 | 1 |  |
|  |  | 4 |  |  |  | 428.4 | 19.7164 | 5 | 367.6 | 3.9853 | 188 |  |  |  |  |
|  |  | mTHI-class |  | | | | | | | | | | | | < 0.001 |
|  | 56 d p.p. | < 40 | 375.0 | 2.8604 | 468 | 380.6 | 8.5017 | 30 |  |  |  | 378.0 | 3.923 | 189 |  |
|  |  | 40 - 49 | 385.5 | 3.7997 | 196 | 375.4 | 3.4105 | 269 | 363.1 | 14.2292 | 11 | 380.2 | 3.4232 | 261 |  |
|  |  | 50 - 59 | 397.5 | 60.8083 | 1 | 368.3 | 2.9442 | 444 | 367.7 | 3.7275 | 214 | 362.5 | 5.5 | 77 |  |
|  |  | ≥ 60 |  |  |  | 391.1 | 3.8934 | 210 | 370.2 | 3.1303 | 441 |  |  |  |  |
|  |  | nHS-class |  | | | | | | | | | | | | < 0.001 |
|  | 56 d p.p. | 1 | 378.6 | 2.5849 | 665 | 370.1 | 2.8598 | 493 | 383.5 | 5.9945 | 64 | 377.8 | 2.851 | 517 |  |
|  |  | 2 |  |  |  | 369.7 | 4.0188 | 166 | 368.5 | 5.1373 | 94 | 377.9 | 16.1914 | 8 |  |
|  |  | 3 |  |  |  | 388.7 | 3.9734 | 184 | 361.3 | 5.0566 | 95 | 343.7 | 30.6719 | 2 |  |
|  |  | 4 |  |  |  | 389.7 | 4.897 | 107 | 372.5 | 3.7735 | 227 |  |  |  |  |
|  |  | 5 |  |  |  | 374.9 | 25.7079 | 3 | 368.2 | 3.9979 | 186 |  |  |  |  |

^1^Traits: BWT = birth weight; 200dg = 200 d-weight gain; 365dg = 365 d-weight gain; CINT = calving interval.

^2^mTHI = mean daily temperature humidity index.

^3^nHS = number of heat stress days.

^4^LSMeans = Least-squares means.

^5^SE = Standard error.

^6^n = number of observations.
