# Supplementary material for: Time-lagged and acute impact of heat stress on production and fertility traits in the local dual-purpose cattle breed “Rotes Höhenvieh” under pasture-based conditions
Source: Transl Anim Sci. 2020 Aug 5;4(3):txaa148. doi: 10.1093/tas/txaa148 (PMC7528550; doi:10.1093/tas/txaa148)
Supplement: txaa148_suppl_Supplementary_Table_S3 [file txaa148_suppl_supplementary_table_s3.docx]

**Supplemental Meterial**

**Table S3:** Effects of mTHI-class or nHS-class and of calving season during the different recording periods for

HS indicators on the probability of stillbirth (SB). The table includes probability of stillbirth (%) with

corresponding SE, number of observations (n) and P-value.

| Recording period for HS indicators | Model | Fixed effect | Trait^1^ | | | |
| --- | --- | --- | --- | --- | --- | --- |
|  |  |  | SB, % | | | |
|  |  |  | % | SE^4^ | *n*^5^ | *P*-value |
| 7 d a.p. | Model 3 | mTHI^2^-class |  | | | > 0.05 |
|  |  | < 40 | 4.90 | 0.7845 | 2,492 |  |
|  |  | 40 - 49 | 3.50 | 0.5460 | 2,246 |  |
|  |  | 50 - 59 | 3.72 | 0.5700 | 2,597 |  |
|  |  | ≥ 60 | 4.68 | 0.8443 | 1,956 |  |
|  |  | calving season |  | | | < 0.05 |
|  |  | winter | 3.78 | 0.6920 | 2,041 |  |
|  |  | spring | 4.61 | 0.6423 | 3,106 |  |
|  |  | summer | 3.17 | 0.5737 | 2,273 |  |
|  |  | autumn | 5.40 | 0.8215 | 1,871 |  |
|  | Model 4 | nHS^3^-class |  | | | > 0.05 |
|  |  | 1 | 4.12 | 0.5015 | 7,080 |  |
|  |  | 2 | 4.45 | 0.8002 | 1,431 |  |
|  |  | 3 | 4.28 | 1.0200 | 780 |  |
|  |  | calving season |  | | | > 0.05 |
|  |  | winter | 4.32 | 0.8161 | 2,041 |  |
|  |  | spring | 4.49 | 0.7530 | 3,106 |  |
|  |  | summer | 3.32 | 0.5211 | 2,273 |  |
|  |  | autumn | 5.20 | 0.9165 | 1,871 |  |
| 42 d a.p. | Model 3 | mTHI-class |  | | | > 0.05 |
|  |  | < 40 | 4.23 | 0.7126 | 2,627 |  |
|  |  | 40 - 49 | 3.49 | 0.5528 | 2,271 |  |
|  |  | 50 - 59 | 3.99 | 0.6063 | 2,855 |  |
|  |  | ≥ 60 | 5.31 | 1.0090 | 1,540 |  |
|  |  | calving season |  | | | < 0.05 |
|  |  | winter | 4.23 | 0.8082 | 2,041 |  |
|  |  | spring | 4.74 | 0.6922 | 3,106 |  |
|  |  | summer | 3.00 | 0.5369 | 2,275 |  |
|  |  | autumn | 5.13 | 0.7985 | 1,871 |  |
|  | Model 4 | nHS-class |  | | | > 0.05 |
|  |  | 1 | 4.13 | 0.5214 | 6,588 |  |
|  |  | 2 | 3.12 | 0.6757 | 1,124 |  |
|  |  | 3 | 4.15 | 0.9153 | 897 |  |
|  |  | 4 | 5.69 | 1.2510 | 684 |  |
|  |  |  |  |  |  |  |
|  |  | calving season |  | | | > 0.05 |
|  |  | winter | 4.12 | 0.8290 | 2,041 |  |
|  |  | spring | 4.41 | 0.8147 | 3,106 |  |
|  |  | summer | 3.20 | 0.5082 | 2,275 |  |
|  |  | autumn | 5.16 | 0.8389 | 1,871 |  |
| 56 d a.p. | Model 3 | mTHI-class |  | | | > 0.05 |
|  |  | < 40 | 4.09 | 0.6935 | 2,627 |  |
|  |  | 40 - 49 | 3.68 | 0.5717 | 2,356 |  |
|  |  | 50 - 59 | 3.93 | 0.6129 | 2,832 |  |
|  |  | ≥ 60 | 5.37 | 1.0070 | 1,478 |  |
|  |  | calving season |  | | | < 0.05 |
|  |  | winter | 4.40 | 0.8143 | 2,041 |  |
|  |  | spring | 4.73 | 0.7041 | 3,106 |  |
|  |  | summer | 3.05 | 0.5407 | 2,275 |  |
|  |  | autumn | 4.99 | 0.7866 | 1,871 |  |
|  | Model 4 | nHS-class |  | | | > 0.05 |
|  |  | 1 | 4.12 | 0.5358 | 6,204 |  |
|  |  | 2 | 3.22 | 0.6974 | 1,053 |  |
|  |  | 3 | 3.33 | 0.8491 | 758 |  |
|  |  | 4 | 4.67 | 1.0490 | 698 |  |
|  |  | 5 | 6.07 | 1.3330 | 580 |  |
|  |  | calving season |  | | | > 0.05 |
|  |  | winter | 4.17 | 0.8569 | 2,041 |  |
|  |  | spring | 4.41 | 0.8458 | 3,106 |  |
|  |  | summer | 3.21 | 0.5058 | 2,275 |  |
|  |  | autumn | 5.11 | 0.7946 | 1,871 |  |

^1^Trait: SB = probability for stillbirth.

^2^mTHI = mean daily temperature humidity index.

^3^nHS = number of heat stress days.

^4^SE = Standard error.

^5^n = number of observations.
